# The Relationship Between Nutritional Status, Micronutrient Deficiency, and Disease Activity in IBD Patients: A Multicenter Cross-Sectional Study

**DOI:** 10.3390/nu17162690

**Published:** 2025-08-20

**Authors:** Marco Valvano, Susanna Faenza, Fabio Cortellini, Antonio Vinci, Fabio Ingravalle, Mauro Calabrò, Lorenza Scurti, Mariagiulia Di Nezza, Sergio Valerio, Angelo Viscido, Giovanni Latella

**Affiliations:** 1 Gastroenterology, Hepatology and Nutrition Division, Department of Life, Health and Environmental Sciences, University of L’Aquila, 67100 L’Aquila, Italy; susfae10@gmail.com (S.F.); mauro.calabro@graduate.univaq.it (M.C.); lorenza.scurti@graduate.univaq.it (L.S.); mariagiulia.dinezza@graduate.univaq.it (M.D.N.); angelo.viscido@univaq.it (A.V.); giovanni.latella@univaq.it (G.L.); 2 Division of Gastroenterology, Galliera Hospital, 16128 Genoa, Italy; 3 Gastroenterologia Ed Endoscopia Digestiva, AUSL Romagna, Ospedale Infermi, 4792 Rimini, Italy; cortellinifabio@gmail.com; 4 Azienda Regionale Emergenza Sanitaria ARES 118, 00149 Rome, Italy; 5 Doctoral School of Nursing Sciences and Public Health, University of Rome “Tor Vergata”, 00133 Rome, Italy; fabio.ingravalle@gmail.com; 6 Alimenta–Studio Nutrizione e Benessere, 71121 Foggia, Italy; ser.valerio9@gmail.com

**Keywords:** inflammatory bowel disease, nutrition, micronutrients, vitamin D, iron, ferritin

## Abstract

Background and aim: Inflammatory bowel diseases (IBD) are chronic conditions that affect the gastrointestinal tract. The chronic inflammatory state promotes a catabolic environment that contributes to undernutrition, while mucosal damage often impairs nutrient absorption. The aim of this study is to evaluate the relationship between nutritional status—including micronutrient deficiencies—and clinical as well as laboratoristics disease activity in a cohort of patients with IBD. Methods: This is a cross-sectional study conducted across three care centers in Italy. Baseline data, clinical disease activity, and laboratory test results were collected. Micronutrient evaluation included measurements of iron, ferritin, vitamin B12, vitamin D, and folate. In addition, hemoglobin and albumin levels were assessed. Pearson correlation analysis was performed to explore the relationship between disease activity and nutritional status. Additionally, receiver operating characteristics (ROC) analysis were performed to identify patients with active diseases. Results: 110 IBD patients (40 Crohn Disease; 70 Ulcerative Colitis) were included. The serum level of Hb, iron, ferritin and vitamin D was different among the active and inactive group (*p*: 0.007; *p*: 0.001; *p*: 0.005; *p*: 0.003) while no difference was found among the other micronutrients evaluated (folic acid, vitamin B12) and albumin. Iron and vitamin D levels demonstrated the highest accuracy in the ROC analysis, with Area Under the Curve (AUC) of 0.76 (*p* < 0.001) and 0.68 (*p* = 0.013), respectively. Vitamin D and Ferritin showed the better performance (based on calprotectin levels). However, their AUC were sub-optimal (AUC 0.68; *p* < 0.001; AUC 0.66; *p* = 0.19. Conclusions: Hemoglobin, iron, ferritin, and vitamin D were associated with disease activity status. However, despite this correlation, their accuracy in discriminating between active and inactive disease appeared to be suboptimal. Folic acid, vitamin B12, and albumin showed poor concordance with disease activity status.

## 1. Introduction

Inflammatory bowel disease (IBD), encompassing Crohn’s disease (CD) and ulcerative colitis (UC), are chronic, debilitating autoimmune disorders characterized by periods of disease activity and remission [1,2]. The incidence of IBD has been steadily increasing over the years, with prevalence more than doubling, reaching nearly 872 cases per 100,000 individuals by 2020 [3,4]. While the exact etiopathogenesis of IBD remains unclear, a complex interplay of environmental, microbial, and genetic factors is believed to contribute to its development, with immune system dysregulation playing a central role. This dysregulation leads to the overexpression of pro-inflammatory cytokines such as IL-6, TNF-α, IL-12, IL-23, and IFN-γ, which sustain chronic intestinal inflammation [5,6]. Both CD and UC share common clinical manifestations, including abdominal pain, weight loss, diarrhea, blood in the stool, and fatigue [7,8]. However, these conditions differ in terms of their location and nature of inflammation. In CD, any part of the gastrointestinal tract can be affected, with a predilection for the distal ileum, and inflammation typically appears as segmental, asymmetrical, and transmural, often leading to complications like fistulas and abscesses [8].

UC, on the other hand, is confined to the colon and rectum and involves only the mucosal layer of the intestine [7,9].

Beyond immunological and genetic components, a role may also be played by contextual factors in influencing disease course and patient outcomes. Chronic conditions are known to intersect with socioeconomic conditions and can, in turn, impact disease management and recovery. For instance, evidence from other chronic disease populations has shown that illness-related disability and treatment burden may contribute to adverse social outcomes, including reduced occupational participation, with potential implications for long-term health and well-being. In IBD, where disease flares, fatigue, and nutritional impairments are common, such dynamics may impact both clinical and quality-of-life outcomes [10,11].

Both CD and UC, due to their chronic and relapsing nature, can lead to severe complications and significantly impair patients’ quality of life [12]. Consequently, considerable efforts have been made to develop therapeutic strategies aimed at improving clinical outcomes [13].

Historically, treatment options have evolved from a lack of targeted therapies to the introduction of conventional drugs such as 5-ASA compounds, corticosteroids, and immunomodulators. More recently, the therapeutic landscape has expanded with the advent of biologic therapies targeting key inflammatory mediators, including anti-TNFα, anti-integrin α4β7, anti-IL-12/23, and anti-IL-23, as well as small molecules like JAK inhibitors and S1P receptor modulators [14].

Treatment goals have also evolved, now including a “treat-to-target” approach, which seeks to modify the disease course and improve long-term outcomes [15,16].

Given the complex and multifaceted nature of IBD, effective management requires a multidisciplinary approach, with personalized assessments tailored to each patient [16]. In this context, regular monitoring of nutritional status is crucial, as micronutrient deficiencies are observed in over 50% of patients with IBD [17,18]. There is growing evidence that both poor nutritional status and micronutrient deficiencies negatively impact treatment efficacy and outcomes [19].

Malnutrition in IBD is a multifactorial condition resulting from reduced dietary intake, impaired nutrient absorption, and increased energy expenditure that is not compensated by nutritional intake. Malnutrition leads to prolonged hospitalizations, reduced quality of life, poor therapeutic responses, and increased relapse rates [18,20].

Currently, there are no standardized guidelines for nutritional management in these patients. The AGA guidelines on diet and nutritional therapies suggest that a Mediterranean diet may be beneficial for patients with mild to moderate IBD, while enteral nutrition may help induce remission and correct malnutrition in Crohn’s disease patients following surgery [21].

Moreover, whether the micronutrient deficiencies observed in IBD patients are a cause or a consequence of disease activity remains unclear. A meta-analysis demonstrated that patients with IBD had 64% higher OR of vitamin D deficiency compared to controls (OR 1.64; 95% CI 1.30–2.08; I^2^ = 7%; *p* < 0.0001) [22].

Although vitamin D supplementation appears to reduce the risk of clinical relapse in IBD patients—particularly those with Crohn’s disease in clinical remission—the quality of this evidence is still uncertain [23,24].

Similar findings have been reported for iron deficiency and anemia, which are associated with a higher risk of hospitalization and poorer disease control in IBD patients. In contrast, data on other micronutrients remain sparse and inconclusive [25].

Given these ongoing uncertainties, the objective of this study is to investigate whether specific laboratory parameters and nutritional deficiencies are correlated with disease activity in patients with IBD.

## 2. Materials and Methods

The study was designed as a cross-sectional study; all clinical investigations were conducted according to the principles laid down in the Declaration of Helsinki. Ethics approval was issued by the Internal Review Board of the University of L’Aquila–MIRB (protocol number: 12/2023, approval date: March 2023). All patients gave written consent to the anonymous processing of their data. The study was reported according to the Strengthening the Reporting of Observational Studies in Epidemiology (STROBE) Statement [26].

This study was conducted across three tertiary care centers in Italy: The Division of Gastroenterology, Hepatology, and Nutrition, University of L’Aquila (L’Aquila); the Division of Gastroenterology, Galliera Hospital (Genoa); and the Gastroenterology and Endoscopy Unit, Ospedale Infermi (Rimini).

We prospectively included all patients with a confirmed diagnosis of IBD who had a scheduled outpatient visit between April 2024 and September 2024.

Inclusion criteria:

- Adult patients (≥18 years old)
- As per study protocol, no nutritional supplementation was allowed at the baseline scheduled visit
- Established diagnosis of IBD (either CD or UC), according to current guidelines [27]
- Biochemical evaluation performed within two weeks of the outpatient visit
- Written informed consent for data processing

Exclusion criteria:

- Biochemical evaluation performed more than two weeks prior to the outpatient visit

Baseline data collected included sex, age, IBD subtype, Montreal classification, disease duration, clinical disease activity, and laboratory test results, C-reactive protein (CRP). Micronutrient evaluation included measurements of iron, ferritin, vitamin B12, vitamin D, and folate. In addition, hemoglobin and albumin levels were assessed as general indicators of nutritional and inflammatory status. No imputation or manipulation was performed in case of missing data.

The normal reference values and corresponding units for each nutritional parameter evaluated are reported in Supplementary Table S1.

### 2.1. Disease Activity

The included patients were categorized as dichotomous groups as active and inactive disease as follows:

- Clinical activity:

UC: Partial Mayo clinical score (PMS) ≥ 3

CD: Harvey-Bradshaw Index (HBI) ≥ 5

- Active disease based on calprotectin levels≥ 150 mcg/kg

### 2.2. Aim

Our primary objective was to evaluate the mean levels of micronutrients in patients with active versus inactive disease. The two groups were classified based on clinical activity and biochemical evidence of inflammation, as determined by calprotectin levels.

### 2.3. Statistical Analysis

Continuous variables were reported as means with standard deviations (±SD) or median and range in case of skewed distributions. An unpaired two-sample Welch *t*-test was performed to compare the micronutrients mean levels among active and inactive patients. The micronutrients that demonstrated differences between the active and inactive disease groups were included in subsequent analyses.

Pearson correlation analysis was performed to explore the relationship between disease activity and nutritional status. Additionally, receiver operating characteristics (ROC) analysis were performed to determine for identifying patients with active diseases. This was performed twice, for clinical and laboratory evidence of disease activity, respectively.

A multivariate analysis was performed, including the micronutrients that differed between the active and inactive groups, and adjusting for CRP levels.

Statistical analysis was conducted at the Department of Biomedicine and Prevention, University of Rome “Tor Vergata”. MS Excel v.2016 (Microsoft Corporation, USA, 2016) and Stata v.17 (College Station, TX: StataCorp LLC, 2021) were used for calculations and graph creation. Significance level was set at *p* = 0.05 for all inferential analysis.

## 3. Results

At the end of the enrollment period, 110 IBD patients (40 CD; 70 UC) were included. Overall, 25/110 (22%) patients presented clinical activity, 38/110 (34%) showed high value of calprotectin. The baseline characteristics are reported in Table 1.

### 3.1. Micronutrients Serum Level and Disease Activity

The serum level of Hb, iron, ferritin and vitamin D was different among the active and inactive group (*p*: 0.007; *p*: 0.001; *p*: 0.005; *p*: 0.003) while no difference was found among the other micronutrients evaluated (folic acid, vitamin B12) and albumin. The results were reported in Table 2.

**Figure 1:**
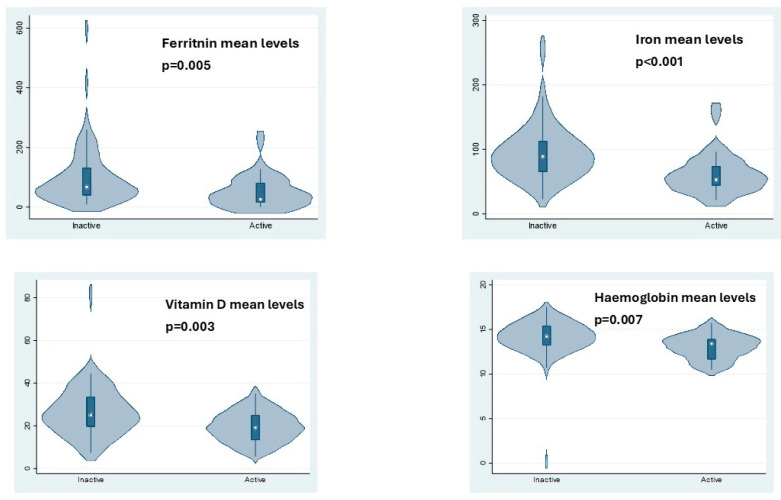
The boxplot shows the mean serum level of ferritin, iron, vitamin D and Hemoglobin among active and inactive groups.

Figure 1 showed the box plot of the mean serum level of Hb, iron, ferritin and vitamin D among the active and inactive groups. Mean serum levels among the active and inactive groups are reported as Supplementary Table S2.

### 3.2. Clinical Activity: Logistic Regressions and ROC Analysis

Hb and the micronutrients that demonstrated differences between the active and inactive disease groups were included in subsequent analyses.

Hb levels showed a suboptimal correlation with clinical disease activity in IBD patients (OR 0.80; 95% CI 0.62–1.03). The ROC analysis yielded an Area Under the Curve (AUC) of 0.70 (*p* = 0.06), suggesting limited discriminatory power. Iron and vitamin D levels demonstrated the highest accuracy in the ROC analysis, with AUCs of 0.76 (*p* < 0.001) and 0.68 (*p* = 0.013), respectively.

**Figure 2:**
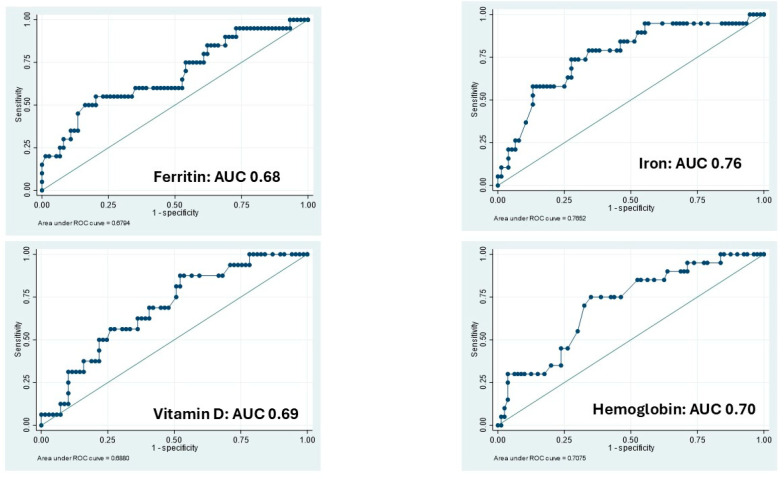
The receiver operating characteristic (ROC) curve shown accuracy of micronutrients and hemoglobin to identify patients with active diseases based on clinical activity. AUC: Area Under the Curve.

Iron levels were associated with an OR of 0.97 (95% CI: 0.95–0.98), while vitamin D levels had an OR of 0.92 (95% CI: 0.86–0.99), indicating a significant inverse association with disease activity (Table 3; Figure 2).

### 3.3. Calprotectin: Logistic Regressions and ROC Analysis

Vitamin D and Ferritin showed the better performance at the ROC analysis considering the disease activity assessed as dichotomic value based on calprotectin levels.

**Figure 3:**
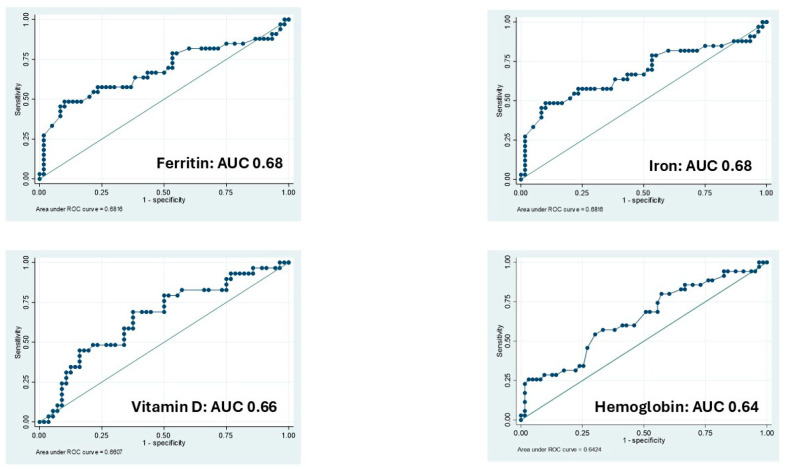
The receiver operating characteristic (ROC) curve shown accuracy of micronutrients and hemoglobin to identify patients with active diseases based on calprotectin value. AUC: Area Under the Curve.

However, their AUC were sub-optimal (AUC 0.68; *p* < 0.001; AUC 0.66; *p* = 0.19) with an OR of 0.98 (95% CI 0.97–0.99) and 0.94 (95% CI 0.89–0.99), respectively (Table 3; Figure 3).

### 3.4. Multivariate Analysis of Micronutrients and Disease Activity

In the multivariate model, which included micronutrients and adjusted for inflammatory status using serum CRP values, the association between micronutrient levels and disease activity was weak and not statistically significant in the multivariate analysis (Table 4).

## 4. Discussion

An increasing body of evidence has highlighted a strong correlation between nutritional status and disease activity in IBD [25]. As a result, the assessment of nutritional status in these patients is considered relevant, and current guidelines recommend routine screening for nutritional status, micronutrient deficiencies, and bone mineral density [17,28].

Deficiencies in nutrients and low serum levels of micronutrients have been associated with poorer outcomes, including reduced quality of life and impaired induction and maintenance of remission [17]. However, existing data are often conflicting and not sufficiently robust to draw definitive conclusions regarding the impact of micronutrient levels on disease course [25].

Moreover, evidence on how micronutrient deficiencies influence disease progression and therapeutic response remains limited [29]. It also remains unclear whether supplementation of specific micronutrients or vitamins, such as vitamin D, acts as a therapeutic intervention or simply reflects disease activity and nutritional depletion secondary to inflammation [24,30].

Conversely, the use of micronutrients and standard biochemical parameters (e.g., leukocyte and platelet counts, hemoglobin levels) as biomarkers of IBD activity has been investigated, but results remain inconsistent [31,32,33,34].

The findings of this study revealed a suboptimal correlation between the evaluated micronutrients and disease activity. It is likely that the outpatient setting contributed to the selection of a patient population with relatively less severe disease and better nutritional status, thereby reducing the observed differences between active and inactive groups. However, this clinical context closely reflects the unselected patient population encountered in routine clinical practice.

Additionally, the relatively small sample size limited the ability to perform subgroup analyses, which may have provided further insights. As reported in other studies, deficiencies in certain micronutrients appear to be more pronounced in patients with CD than in those with UC. In particular, this finding is more robust for vitamin D and vitamin B12, especially in CD patients who have undergone ileal resection [23,24]. However, the limited sample size in the present study does not allow for sub-analyses to robustly verify this observation.

Conversely, the stringent inclusion criteria, which excluded patients receiving any form of nutritional supplementation, enhance the robustness and reproducibility of our results.

Notably, serum vitamin D levels demonstrated the highest accuracy in discriminating disease activity, both clinical and biochemical, in ROC analyses—with AUCs of 0.68 (*p* = 0.013) and 0.68 (*p* < 0.001), respectively. Furthermore, vitamin D showed a significant inverse association with active disease, with odds ratios of 0.92 (95% CI: 0.86–0.99) for clinical activity and 0.98 (95% CI: 0.97–0.99) for biochemical activity. The data concerning vitamin D appears to be the most robust among the evaluated micronutrients and are consistent with previously published findings in the literature [35].

Previous evidence has suggested a relationship between vitamin D supplementation and disease course in patients with IBD [23]. However, these findings remain conflicting, as highlighted in a recent meta-analysis [24]. In a study involving selected patients receiving biological therapy, only the cut-off of ≥25 mg/dL was evaluated as a meaningful target for IBD patients [34]. Future studies should clearly define the baseline population, the specific purpose of vitamin D treatment, and an appropriate dosing strategy, in order to generate robust evidence and provide clear recommendations regarding vitamin D supplementation.

Regarding serum hemoglobin, mean levels were higher in the inactive group; however, the correlation with disease activity was less pronounced (OR 0.80; 95% CI: 0.62–1.03).

Although ferritin is an acute-phase reactant and its serum levels may be elevated in IBD patients with active inflammation, its association with disease activity appears more reliable in patients with low-grade systemic inflammation, as evidenced by the good correlation observed in the population included in this study. Although differences between the active and inactive groups were consistent for all micronutrients analyzed, some minor discrepancies emerged (e.g., ferritin was associated with calprotectin but not with clinical activity). This discrepancy can be speculatively explained by the weak correlation between clinical activity and endoscopic inflammatory activity.

Although the results presented in this study are consistent with previously published data, two important considerations should be noted. First, in an unselected population of IBD outpatients—receiving both conventional and biological therapies—the differences between active and inactive groups were only modest. Most importantly, the limited magnitude of these differences does not support the use of serum micronutrient levels as reliable biomarkers for identifying disease activity.

Micronutrient deficiencies are more prevalent in hospitalized patients, as well as in those with active IBD and long-standing CD. According to current guidelines, monitoring of micronutrient levels should be considered in people with IBD, and supplementation is recommended in cases of deficiency [19].

## 5. Conclusions

Hemoglobin, iron, ferritin, and vitamin D levels were associated with disease activity status. However, despite these correlations, their accuracy in distinguishing between active and inactive disease was suboptimal, limiting their utility as reliable biomarkers. In contrast, folic acid, vitamin B12, and albumin levels demonstrated poor concordance with disease activity, further reducing their potential clinical relevance in this context.

## Figures and Tables

**Table 1:** Baseline characteristics among active/inactive groups.

	Clinical Active Disease (n = 25)	Clinical Inactive Disease (n = 85)
**Disease**		
CD	9	33
UC	16	52
**Sex gender**		
Male	12	52
Female	13	33
**Therapy**		
Advanced therapies	12	49
Conventional therapies	13	36
**Disease duration (months)**	Median: 88.5 (Range: 2–300)	Median: 96.0 (Range: 6–588)
**BMI**		
**CD**	23.35 (4.41)	24.29 (2.56)
**UC**	24.5 (3.23)	24.63 (2.90)
**Localization**		
**CD**		
Ileal	4	18
Ileo-colic	5	13
Colic	0	2
**UC**		
Ulcerative proctitis	7	19
Left side colitis	5	20
Extensive colitis	4	13

CD: Crohn Disease; UC: Ulcerative colitis; Advanced Therapy: Biologics or small molecules.

**Table 2:** Biochemical mean levels and differences among active and inactive groups, high and low calprotectin level groups, and active and inactive groups.

Item	Missing Data (N)	Mean Difference Among Clinical Active/Inactive Groups (Standard Error)	*p*-Value (95% CI)	Mean (SD)
Hemoglobin	10 (100)	∆ + 1.02 mg/dL (0.39)	*p* = 0.007 (13.45–14.26)	Active: 13.05 (1.42) Inactive: 14.07 (2.13)
Iron	15 (95)	∆ + 33.03 μg/dL (8.82)	*p* < 0.001 (79.46–97.13)	Active: 61.88 (31.73) Inactive: 94.90 (43.55)
Ferritin	16 (94)	∆ + 44.59 ng/dL (16.90)	*p* = 0.005 (70.25–107.68)	Active: 53.87 (56.40) Inactive: 98.46 (96.87)
Vitamin D	25 (85)	∆ + 6.84 mg/dL (2.35)	*p* = 0.003 (2.2.79–27.68)	Active: 19.67 (7.61) Inactive: 26.51 (11.59)
Vitamin B12	57 (53)	∆ + 107.3 pg/dL (102.90)	*p* = 0.16 (−336.72–122.12)	Active: 370.00 (136.62) Inactive: 477.30 (318.67)
Folic Acid	47 (63)	∆ − 1.90 ng/dL (2.98)	*p* = 0.267 (−8.30–4.49)	Active: 8.43 (10.40) Inactive: 6.53 (5.39)
Albumin	45 (65)	∆ + 0.12 g/dL (0.13)	*p* = 0.203 (−0.16–0.39)	Active: 4.12 (4.05) Inactive: 4.23 (3.90)
Calprotectin	11 (99)	∆ − 917.44 μg/gr (285.27)	*p* = 0.002 (186.10–474.20)	Active: 1062.25 (1266.58) Inactive: 144.81 (303.93)
**Item**	**Missing Data** **(N)**	**Mean Difference Among** **Low/High Calprotectin Groups** **(Standard Error)**	***p* Value** **(95% CI)**	**Mean** **(SD)**
Hemoglobin	12 (98)	∆ + 0.79 mg/dL (0.31)	*p* = 0.006 (0.18–1.42)	High: 13.49 (1.60) Low: 14.29 (1.41)
Iron	17 (93)	∆ + 17.04 μg/dL (9.18)	*p* = 0.033 (−1.21–35.29)	High: 77.87 (49.56) Low: 94.91 (37.96)
Ferritin	19 (91)	∆ + 66.32 ng/dL (18.76)	*p* < 0.001 (29.03–103.60)	High: 50.83 (40.98) Low: 117.14 (104.54)
Vitamin D	25 (85)	∆ + 5.51 mg/dL (2.49)	*p* = 0.015 (0.56–10.47)	High: 21.48 (8.72) Low: 26.99 (11.83)
Vitamin B12	59 (51)	∆ + 72.66 pg/dL (52.63)	*p* = 0.08 (−33.09–178.41)	High: 352.09 (123.65) Low: 424.75 (225.85)
Folic Acid	49 (61)	∆ + 0.42 ng/dL (1.77)	*p* = 0.407 (−3.12–3.96)	High: 6.79 (7.76) Low: 7.21 (6.05)
Albumin	48 (62)	∆ + 0.01 g/dL (0.17)	*p* = 0.466 (−0.33–0.36)	High: 4.19 (0.35) Low: 4.21 (0.81)
**Item**	**Missing Data** **(N)**	**Mean Difference Among** **Active/Inactive Groups †** **(Standard Error)**	***p*** **Value** **(95% CI)**	**Mean** **(SD)**
Hemoglobin	8 (102)	∆ + 0.48 mg/dL (0.20)	*p* = 0.106 (−0.28–1.23)	Healthy: 14.04 (2.29) Diseased: 13.56 (1.55)
Iron	13 (97)	∆+ 14.78 μg/dL (9.43)	*p* = 0.061 (−4.06–33.61)	Healthy: 94.23 (38.37) Diseased: 79.45 (48.77)
Ferritin	14 (96)	∆ + 48.66 ng/mL (16.06)	*p* = 0.002 (16.74–80.58)	Healthy: 108.61 (105.09) Diseased: 59.95 (50.66)
Vitamin D	23 (87)	∆ + 5.47 mg/dL (2.25)	*p* = 0.009 (0.98–9.95)	Healthy: 27.24 (11.80) Diseased: 21.77 (9.09)
Vitamin B12	57 (53)	∆ − 5.14 pg/dL (52.83)	*p* = 0.462 (−111.91–101.64)	Healthy: 387.82 (144.51) Diseased: 392.96 (226.11)
Folic Acid	47 (63)	∆ + 0.79 ng/mL (1.72)	*p* = 0.322 (−2.64–4.24)	Healthy: 7.29 (6.22) Diseased: 6.49 (7.25)
Albumin	45 (65)	∆ − 0.01 g/dL (.15)	*p* = 0.469 (−0.31–0.28)	Healthy: 4.20 (1.81) Diseased: 4.22 (0.33)
Calprotectin	9 (101)	∆ − 734.12 μg/gr (156.87)	*p* < 0.001 (−1051.39–416.85)	Healthy: 41.15 (39.15) Diseased: 775.28 (991.61)

∆ = Mean difference; CI: Confidence Interval; SD: Standard Deviation; Calpro: calprotectine; †: Active disease was defined as the presence of either clinical disease activity or an elevated calprotectin level.

**Table 3:** Normal levels of micronutrients and relative Odds Ratio and Area Under the Curve in active group (both clinical and calprotectin activity).

	Clinical Activity		Calprotectin	
Micronutrients and Hemoglobin	Odds Ratio (95% CI)	AUC	Odds Ratio (95% CI)	AUC
Hemoglobin	0.80 (95% CI: 0.62–1.03)	0.70 (*p* = 0.06)	0.69 (95% CI: 0.51–0.93)	0.64; *p* = 0.01
Iron	0.97 (95% CI: 0.95–0.98)	0.76 (*p* < 0.001)	0.98 (95% CI: 0.97–1.00)	0.68; *p* = 0.05
Ferritin	0.98 (95% CI: 0.97–1.00)	0.68 (*p* = 0.018)	0.94 (95% CI: 0.89–0.99)	0.68; *p* = 0.19
Vitamin D	0.92 (95% CI: 0.86–0.99)	0.69 (*p* = 0.013)	0.98 (95% CI: 0.97–0.99)	0.66; *p* < 0.001

CI: Confidence Interval; AUC: Area Under the Curve.

**Table 4:** Multivariate model incorporating micronutrients and disease activity, defined by both calprotectin levels and clinical activity.

Micronutrients and Hemoglobin	Odds Ratio (95% CI)	*p* Value
Hemoglobin	0.75 (0.50–1.12)	0.170
Iron	0.99 (0.98–1.00)	0.655
Ferritin	0.99 (0.92–1.02)	0.082
Vitamin D	0.97 (0.93–1.02)	0.270
CRP	1.07 (0.93–1.24)	0.314

CI: Confidence Interval. CRP: C-reactive protein.

## Data Availability

The datasets generated during and/or analyzed during the current study are available from the corresponding author on reasonable request.

## Supplementary Materials

The following supporting information can be downloaded at: https://www.mdpi.com/article/10.3390/nu17162690/s1, Table S1: normal reference values and corresponding units of measurement; Table S2: Mean serum level (±SD) among active and inactive groups.

## Abbreviations

The following abbreviations are used in this manuscript:

IBD	Inflammatory Bowel Diseases
ROC	Receiver Operating Characteristics
CD	Crohn’s Disease
UC	Ulcerative Colitis
OR	Odds Ratio
PMS	Partial Mayo clinical score
HBI	Harvey-Bradshaw Index
SD	standard deviations
AUC	Area Under the Curve
