# Supplementary material for: The Relationship Between Nutritional Status, Micronutrient Deficiency, and Disease Activity in IBD Patients: A Multicenter Cross-Sectional Study
Source: Nutrients. 2025 Aug 20;17(16):2690. doi: 10.3390/nu17162690 (PMC12389598; doi:10.3390/nu17162690)
Supplement: Supplementary file 1 [file nutrients-17-02690-s001.zip › nutrients-3805970-supplementary.pdf]

Supplementary Table S1: normal reference values and corresponding units of measurement

	**Normal values**
**Hemoglobin (gr/dl)**	Male ≥ 13; Female ≥ 12
**Iron (μg/dl)**	50-170
**Ferritin ng/ml**	4-210
**Vitamin D – 25(OH)D (mg/dl)**	<25
**Vitamin B12 (pg/ml)**	157-1059
**Folic acid (ng/ml)**	4.6-18.7
**Albumin g/dl**	4.02-4.76

Supplementary Table S2: Mean serum level (±SD) among active and inactive groups

	**Inactive group (±SD)**	**Active group (±SD)**
**Hemoglobin**	14.06 (2.13)	13.05 (1.4)
**Iron**	94.90 (43.54)	61.87 (31.72)
**Ferritin**	98.45 (96.87)	53.87 (56.40)
**Vitamin D**	26.51 (11.59)	19.66 (7.60)
**Vitamin B12**	370 (136.61)	477.3 (318.66)
**Folic acid**	6.52 (5.38)	8.42 (10.40)
**Albumin**	4.23 (0.69)	4.11 (0.35)
